# Spatiotemporal Remodeling of Presynaptic Terminals in Human Neuromuscular Junctions

**DOI:** 10.3390/ijms27041928

**Published:** 2026-02-17

**Authors:** Zhanyang Liang, Sebastian L. Schubert, Aline Müller, Miguel Pishnamaz, Frank Hildebrand, Mahtab Nourbakhsh, Xiaoying Chen

**Affiliations:** 1 Institute of Pathology, RWTH Aachen University Hospital, 52074 Aachen, Germany; zliang@ukaachen.de (Z.L.); almueller@ukaachen.de (A.M.); 2 Clinic for Orthopedics, Trauma, and Reconstructive Surgery, RWTH Aachen University Hospital, 52074 Aachen, Germany; seschubert@ukaachen.de (S.L.S.); mpishnamaz@ukaachen.de (M.P.); fhildebrand@ukaachen.de (F.H.)

**Keywords:** human, skeletal muscle, tissue model, neuromuscular junction, SV2, 2H3, presynaptic terminal, tissue-resident macrophages, fatty acid

## Abstract

Neuromuscular junctions (NMJs) connect motor neurons to muscle fibers, enabling electrical-to-chemical signal transmission and sensing of mechanical forces. We employed a previously introduced human skeletal muscle tissue model to study the NMJ presynaptic terminal (PT). Using immunohistochemical approaches, we analyzed PT formation in samples from 12 participants, either immediately after surgery or following 11 days of in vitro maintenance. In immediate postsurgical samples, the number, size, and integrated immunofluorescence intensity of detectable PTs increased with participant age, accompanied by elevated expression of neurotrophic and inflammatory proteins. Moreover, CD80^+^ and CD206^+^ macrophages were detected near PTs, suggesting a potential regulatory role in PT formation. Prolonged in vitro maintenance of muscle samples in a quiescent state for 11 days reproduced age-associated PT features and further increased PT spatial expansion and fragmentation. Notably, increased PT fragmentation positively correlated with participant body mass index (BMI), suggesting a possible link between metabolic status and PT remodeling. Consistently, the activation of skeletal muscle metabolism through long-chain fatty acid supplementation partially restored PT formation in vitro. Together, these findings highlight the utility of human skeletal muscle tissue models for investigating NMJ dynamics in pathological contexts and for identifying therapeutic strategies aimed at delaying or preventing neuromuscular decline.

## 1. Introduction

Neuromuscular junctions (NMJs) are specialized chemical synapses between motor neurons and skeletal muscle fibers. Their structural and functional integrity is essential for maintaining motor control and overall neuromuscular function. Three key functional domains of the NMJ were defined previously: the presynaptic neuron terminal (PT) with synaptic vesicles, the synaptic cleft with extracellular matrix (ECM), and the postsynaptic junctional folds of muscle fibers with clustered acetylcholine receptors (AChRs). Together, they convert electrical impulses generated by motor neurons into electrical activity in muscle fibers. PTs release acetylcholine, while the postsynaptic membrane detects acetylcholine via AChRs and triggers depolarization. Multiple lines of evidence indicate that NMJs also exhibit mechanosensitivity via different mechanisms [1]. For instance, Piezo Type Mechanosensitive Ion Channel Component 1 (Piezo 1), a mechanosensitive channel expressed in skeletal muscle fibers and neurons, has been shown to support neuron formation and increase Ca^2+^ influx, which may affects AChR clustering and NMJ activity [2,3,4]. Mechanical tension has been reported to maintain NMJ integrity and AChR clustering through direct activation of ECM integrin signaling [5,6,7]. Recent functional studies have also revealed that exercise preserves NMJ morphology in aging skeletal muscles, suggesting that mechanotransduction at NMJ is crucial for maintaining skeletal muscle activity [8]. In addition, NMJs are surrounded by different types of cells with regulatory roles, including maintenance of synaptic structure, and early damage sensing. Among these, Schwann cells are the best characterized NMJ-associated cells and play a crucial role in the development and maintenance of NMJs [9,10].

A number of important structural, functional, and physiological differences were found between human and mouse NMJs [11,12]. Compared with mouse NMJ, human NMJ is smaller but highly efficient and structurally complex [12]. They exhibit high AChR density and deep junctional folds, increasing the postsynaptic surface area [11]. These characteristics likely compensate for the smaller synaptic area. However, the compact architecture of human NMJs may increase the vulnerability to aging and diseases, such as amyotrophic lateral sclerosis (ALS) and sarcopenia [13,14,15]. In mice, neuromuscular junctions (NMJs) are known to undergo age-associated degeneration, which is accompanied by decreased metabolic activity in skeletal muscle. [16]. Notably, neuronal cells are highly vulnerable to impaired metabolism, leading to ATP deficits and reduced synaptic vesicle release and NMJ activity [17]. These findings highlight the essential role of metabolism in maintaining NMJ function.

Tissue-resident macrophages (TRMs) are a diverse, self-renewing population of immune cells that maintain tissue homeostasis. They dynamically alter their localization, morphology, and functional properties in response to injury and disease [18,19]. Previous studies of bone marrow derived macrophages have further established a general classification into two major phenotypic states—proinflammatory (M1) and anti-inflammatory (M2)—across humans and other species [20]. However, reports within the past decade have established that most tissue-resident macrophages (TRMs) in both mice and humans originate during embryogenesis and are maintained largely independent of bone marrow-derived monocytes [21]. Furthermore, increasing evidence has indicated that the M1/M2 paradigm oversimplifies the wide spectrum of functional states observed in vivo, particularly among TRMs [22]. Although a universal TRM marker remains unidentified, several markers, including CD80 and CD206, have been verified in multiple human and animal tissues. Recent animal studies have suggested that TRMs may contribute to peripheral neuropathies and the degeneration of NMJs and skeletal muscle [23,24]. Moreover, macrophage depletion in mice was reported to ameliorate age-related degenerative changes in peripheral nerves [25].

Evaluating human NMJ morphology, particularly PT remodeling, is critical for elucidating disease progression and for accurately assessing the efficacy of therapeutic interventions. PT plasticity has been investigated predominantly in animal models, particularly in rodents, and few human studies have been conducted [26,27,28,29,30]. Two well-established antibodies are typically used to label specific PT protein markers—neurofilament (2H3) and synaptic vesicle protein (SV2)—in paraffin-embedded tissue sections, which offer high structural stability. In contrast, postsynaptic AChRs can be visualized only after rapid or repeated freezing of tissue sections and binding to specific antibodies or bungarotoxin [31]. Nevertheless, studying human NMJ morphology remains challenging, largely because of ethical constraints and the limited availability of experimental models that accurately recapitulate human tissue architecture and function. Previously, we developed an **in vitro** model of human skeletal muscle tissue derived from surgical specimens [32]. By maintaining skeletal muscle tissue in vitro, this model preserves diverse cellular populations and functional structures, providing a valuable platform for studying cellular interactions within the native microenvironment of human skeletal muscle. Initially, we applied this model to study the effects of different species of fatty acids (FAs) on the metabolic activity of muscle fibers, resident macrophages [12], and stem cells in the native environment of human skeletal muscle tissue [33]. We previously reported that long-chain FAs activate the metabolic activity and specific subtypes of TRMs in human skeletal muscle tissue in vitro [34]. Here, we employed this study model to investigate PT formation and remodeling in human skeletal muscle tissue under different metabolic conditions. Despite the limited number of donors (*n* = 12), we identified significant correlations between PT characteristics and age, body mass index (BMI), expression levels of neurotrophic and inflammatory proteins, and the presence of proximate TRMs. Moreover, we evaluated the impact of FA-stimulated metabolic activity on PT remodeling in vitro over 11 days, supporting a positive link between metabolism and PT integrity.

## 2. Results

**Figure 1:**
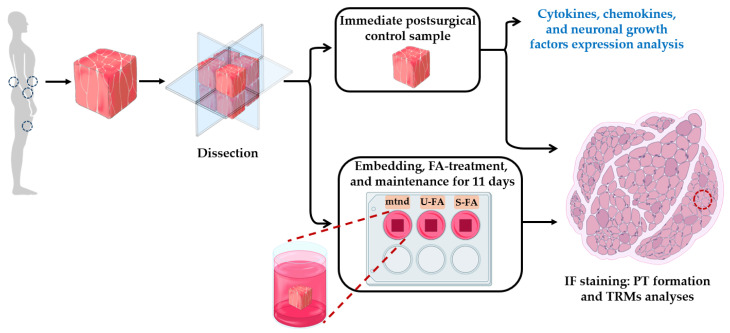
Schematic representation of the experimental study approach.

Human neuromuscular junctions (NMJs) and their interactions with auxiliary cells remain insufficiently studied, largely because of the ethical and logistical challenges associated with their investigation. Here, we used a previously established ex vivo model of human skeletal muscle tissue to examine spatiotemporal changes in the PT of NMJs within their native environment (Figure 1).

We included native skeletal muscle specimens from 12 medically fit adults who underwent surgical treatment and provided written informed consent to donate their excised tissues for experimental use. No other exclusion criteria were applied. The most relevant characteristics of all the study participants are summarized in Table 1. The group of participants, although small, they represent diverse characteristics. The ages of the participants in the study ranged from 22 to 84 years; 6 females and 6 males, including a female diagnosed with type 2 diabetes (T2D) were included. The body mass index (BMI) of the study participants ranged between 19.4 and 46.9 kg/m^2^. All the donors were evaluated as medically fit before the surgeries.

### 2.1. Analysis of PT in a Human Skeletal Muscle Ex Vivo Model

Previous animal studies have suggested that NMJ impairment, whether caused by toxins, injuries, or genetic mutations, is accompanied by PT fragmentation and remodeling [35,36,37]. These observations suggest that similar conformational alterations occur in human skeletal muscle PT; however, experimental confirmation is lacking. We studied possible PT alterations under experimental conditions in human skeletal muscle model (Figure 1). Therefore, skeletal muscle tissue samples were prepared to obtain multiple equal sections from each study participant. One section was designated as an immediately postsurgical control (ctrl). Other sections were maintained in vitro for 9 days (mtnd) with or without saturated or unsaturated FAs (S-FA or U-FA, respectively). FAs are thought to regulate metabolism and activate tissue-resident macrophages (TRMs), potentially modulating PT remodeling [33].

**Figure 2:**
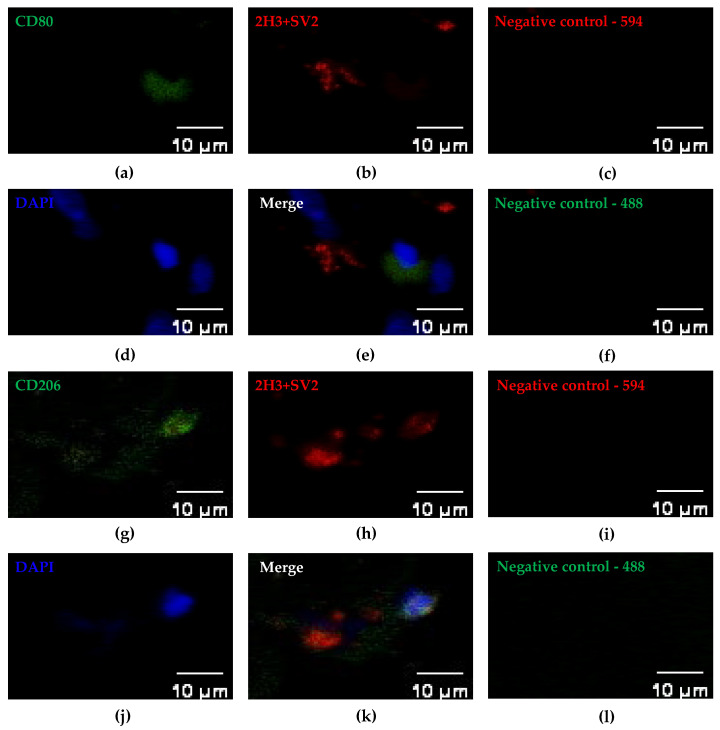
Representative IF images of immediate postsurgical skeletal muscle tissue. The tissue sections were subjected to immunofluorescence staining for neurofilament 2H3 and synaptic vesicle protein SV2 (red) together with DAPI (blue) and CD80 (green; (**a**,**b**,**d**,**e**)) or CD206 (green; (**g**,**h**,**j**,**k**)). Fluor 488-conjugated secondary antibody (green) and Fluor 594-conjugated secondary antibody (red) were used to detect PTs and macrophages, respectively. Staining without antibodies against 2H3 and SV2 (**c**,**i**) or CD80 or CD206 (**f** and **l**, respectively) served as negative controls. Images of the same field were obtained using DyLight 488 (**a**,**b**,**d**) or 594 (**g**,**h**,**j**) filters and then merged to facilitate clearer recognition of labeled structures (**e**,**k**). The white scale bars (lower right) indicate 10 µm, and IF images correspond to tissue fields of 50 µm × 30 µm.

To ensure consistent PT analyses across tissue specimens collected throughout the present study, we used paraffin embedding, which provides superior structural preservation and is well suited for long-term investigations. Frozen sections allow simultaneous imaging of postsynaptic terminals (Supplementary Materials Figure S1); however, they are less stable and unsuitable for long-term preservation. We analyzed all the collected paraffin sections simultaneously using immunofluorescence staining. Two previously established PT markers—neurofilament (2H3) and synaptic vesicle protein (SV2)—were used for the detection of PTs in a series of human skeletal muscle specimens. In addition, antibodies against macrophage markers, CD80 or CD206, together with DAPI nuclear staining were used to profile macrophages that may reside close to PTs. Fluor 488-conjugated and Fluor 594-conjugated secondary antibodies were used to detect the primary antibodies. The sets of representative images are shown in Figure 2. Negative controls lack primary antibodies (Figure 2c,f,i,l), confirming the specificity of detected signals.

To enable a detailed quantitative analysis of the PT images, we defined three independent variable sets, which are summarized in Table 2. The primary variables—including the number of detectable PTs and their cumulative (total PT size) or average area site (avg. PT size) within a view field of ~0.252 mm^2^—capture the overall PT distribution within each sample group. The secondary variables quantify integrated IF densities (IntDen), thereby reflecting the aggregate expression levels of SV2 and 2H3. The tertiary variables characterize the spatial organization of PTs within the sample set, including fragmentation index (FI), circularity index (CI), Roundness index (RI), aspect ratio (AR), and solidity index (SI). All the variables were subsequently evaluated across more than 1900 images acquired from control (cntrl) and maintained (mtnd) tissue samples from all the participants.

### 2.2. Primary and Secondary PT Variables Exhibit Significant Correlations with Participant Age

**Figure 3:**
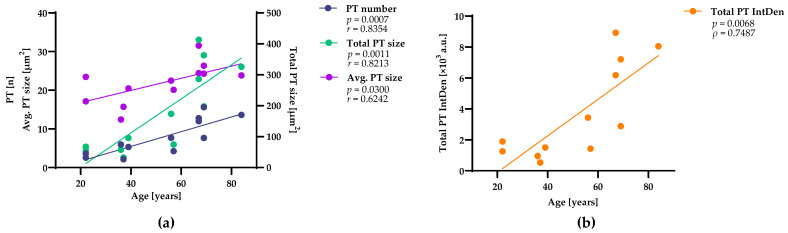
The PT image variables correlated with participant age. Tissue samples (*n* = 12) were subjected to immunofluorescence staining for the detection of PT. Pearson correlation and Spearman’s rank correlation analyses were used to determine significant relationships between the PT image variables (*y*-axes) and participant age (*x*-axes). (**a**) Dot plot shows the correlations between the PT number (n), average size (µm^2^), or total size (µm^2^) and participant age (years, *x*-axis). (**b**) Dot plot shows the correlations between the PT integrated IF density (arbitrary unit, a.u.) and participant age (years, *x*-axis). The calculated correlation coefficients (*r*, *ρ*) and significance levels (*p*) are presented at the top right of the plots. Representative IF images from samples across different age groups were provided in Supplementary Materials Figure S2.

To assess potential PT alterations associated with aging in human skeletal muscle, we conducted correlation analyses between PT variables measured in immediate postsur-gical control samples (ctrl) and participant age. We observed significant age-related in-creases in PT number as well as in their average and cumulative sizes (Figure 3a). Similarly, the summed integrated densities of the SV2 and 2H3 IF signals increased significantly with age (Figure 3b). Notably, the magnitude and statistical significance of the detected correlations remained remarkably high despite the limited sample area. No significant correlations were found between PT characteristics and the anatomical location of the samples (*p ≤* 0.0559). These findings suggest that the primary variables examined in this study may have potential as markers for PT assessment in humans.

### 2.3. Tissue-Resident Macrophages (TRMs) Are Positioned Close to Human PTs

In human skeletal muscle specimens, CD206 and CD80 are the most frequently validated markers used to identify TRMs [38]. To investigate whether TRMs colocalize with PTs in human muscle tissue, we first attempted to quantify their spatial relationship by subjectively estimating the distance from each TRM to the nearest PT. Previous studies estimated a mean PT diameter of 36 µm [13]. Using 50% of the mean PT diameter as an arbitrary cutoff, we assessed the number of CD80^+^ or CD206^+^ TRMs positioned within 18 µm distance to the closest PT border. The number of detected PTs in all the samples was normalized to the examined tissue area (~0.252 mm^2^). As summarized in Table 3, more than 5% of the PTs were associated with CD80^+^ TRMs, and more than 8% were associated with CD206^+^ TRMs. Because CD80^+^ and CD206^+^ cells constitute only a small fraction of TRM populations in human skeletal muscle, these findings suggest that macrophages may represent a stable cellular PT constituent in NMJs.

### 2.4. PT Variables Are Correlated with the Expression of Neurotrophic and Inflammatory Proteins in Human Skeletal Muscle Tissue

**Figure 4:**
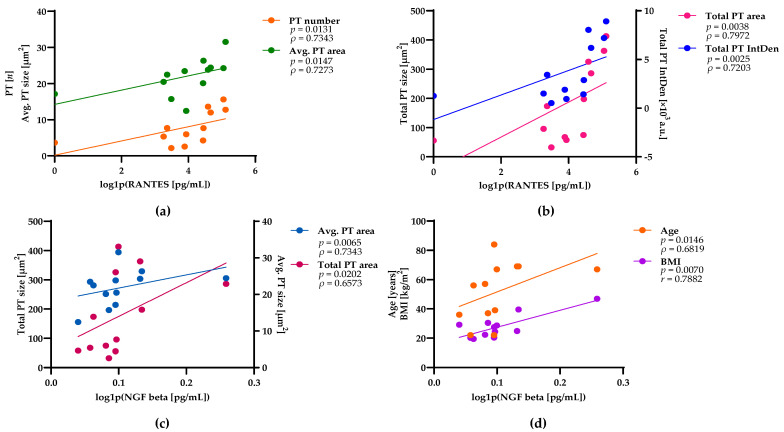
PT characteristics is correlated with RANTES and NGF beta expression in human skeletal muscle tissue. Tissue samples (*n* = 12) were subjected to multiplex protein assays and immunofluorescence staining for the detection of PT variables, as described in the legend to Figure 3. After log transformation of the data, Pearson correlation and Spearman’s rank correlation analyses were used to determine significant relationships between the log-transformed RANTES or NGF beta expression in human skeletal muscle tissue samples (*x*-axes) and PT image variables (*y*-axes). Dot plots show the correlations of log transformed RANTES expression with the PT number and avg. size (**a**) or total size (µm^2^) and integrated density (IntDen) (**b**). Dot plot shows the correlations of log NGF beta expression (*x*-axes) with PT average (avg.) and total sizes (**c**), or with BMI (kg/m^2^) and participant age (years, *y*-axis) (**d**). The calculated correlation coefficients (*r*, *ρ*) and significance levels (*p*) are presented at the top right of the plots.

Understanding the crosstalk between NMJs and macrophages may play an important role in restoring NMJ function after injury. We aimed to assess the possible link between the expression levels of neurotrophic and inflammatory proteins and the PT variables in human skeletal muscle samples. The neurotrophic expression profile in human skeletal muscle tissue has not been reported or studied previously. We applied a multiplex approach for the detection of 4 neurotrophic factors (BDNF, CNTF, GDNF, and NGF beta) and 16 cytokines/chemokines (eotaxin, GRO alpha, IFN alpha, IL-1 alpha, IL-1RA, IL-7, IL-8, IL-15, IL-31, IP-10, MCP-1, MIP-1 alpha, MIP-1 beta, RANTES, SDF-1 alpha, and TNF beta). All samples were analyzed in two independent runs, and the resulting mean concentrations among multiple proteins and samples followed nonnormal distribution. Therefore, we applied the log transformation to conform the data and increase the validity of the statistical analyses. Among the 20 analyzed proteins, RANTES and NGF beta tissue expression levels were significantly correlated with PT number, size, and total IntDen, as shown in Figure 4a,b,c, respectively. The other 18 proteins showed no significant correlations with PT image parameters. These results suggest that RANTES and NGF beta are associated with the PT formation. Interestingly, NGF beta expression in human muscle tissue increased with age and BMI. These correlations were consistent with age related PT characteristics (Figure 4d) and indicated the important role of NGF beta.

### 2.5. Increased PT Expansion in Human Skeletal Muscle Tissue Model

**Figure 5:**
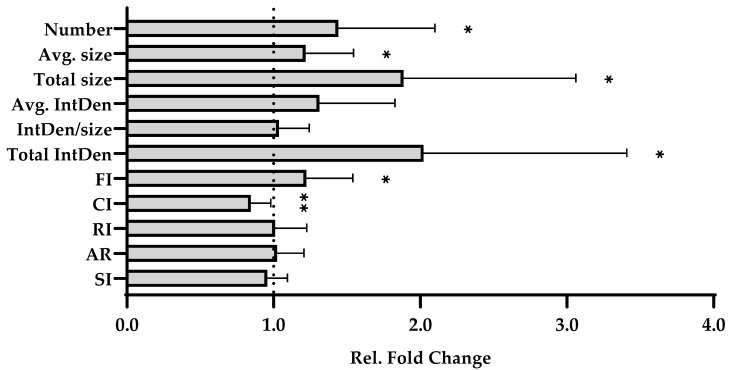
PT remodeling in human skeletal muscle tissue in vitro. Skeletal muscle tissue sections from all participants (*n* = 12) were analyzed either immediately after collection (ctrl) or following in vitro maintenance (mtnd). After IF staining and imaging, PT images of the mtnd group were compared to their respective ctrl images according to the specified variables (*y*-axis), including the number of PTs (number), average size of PTs (avg. size), total size of PTs (total size), integrated density of PT IF integrated density (IntDen), IntDen normalized to PT size (IntDen/size), total IntDen (total IntDen), fragmentation index (FI), circularity index (CI), roundness index (RI), aspect ratio (AR), and solidity index (SI). To determine the relative fold changes (*x*-axis), PT variables of the mtnd images were normalized to their respective ctrl images, which were set to 1 as a reference (dotted line). A paired *t*-test or Wilcoxon signed-rank test was used to assess the significance of the detected differences. The corresponding data are presented in the Table S1. *p* ≤ 0.05 (*), *p* ≤ 0.01 (**). Representative IF images of control and mtnd tissue samples were provided in Supplementary Materials Figure S3.

To assess potential PT alterations in resting human skeletal muscle, we compared the PT variables obtained from maintained tissue samples (mtnd) with those derived from immediate postsurgical control samples (ctrl). As shown in Figure 5, five PT variables—number, fragmentation index (FI), average size, total size, and integrated density—significantly increased in vitro, whereas the circularity index (CI) significantly decreased. Notably, however, the magnitude of the changes in cumulative PT size and IF density was greater than that of FI or CI. Together, the observed alterations closely resemble PT expansion previously reported in animal injury models [35,36,37].

### 2.6. PT Circularity and Fragmentation Dynamics Conversely Correlate with Increasing BMI

**Figure 6:**
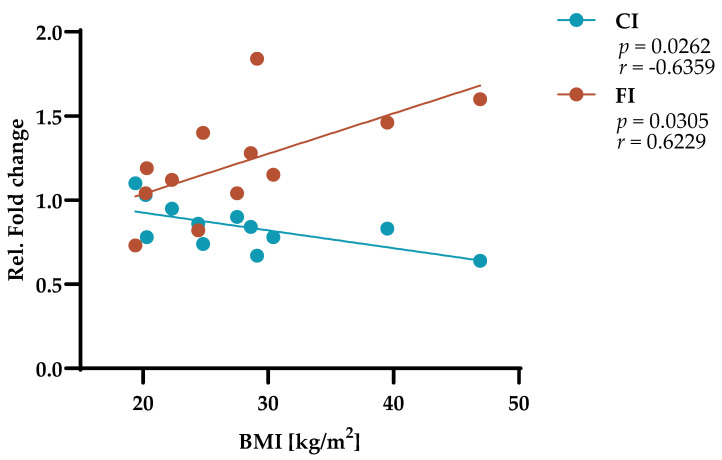
PT circularity index (CI) and fragmentation index (FI) are inversely correlated with increasing BMI. PT IF images were quantified according to the specified variables in Table 2. The relative fold change in PT variables was determined as described in the legend of Figure 5.

To investigate the potential role of different physiological conditions in PT formation, we analyzed the correlations between the determined fold changes in PT variables and different participants’ characteristics, including age, sex, and BMI. Notably, we identified two pronounced and opposing correlations between PT variables and participants’ BMI (Figure 6). First, the significant decrease in PT CI in vitro moderately intensified with increasing BMI. Second, the increase in PT FI in vitro was strongly increased with increasing BMI. The relevance of these data was supported by a high level of statistical significance (*p* < 0.05). Thus, the observed decrease in CI accompanied by increased FI is indicative of PT expansion, with this phenomenon being more pronounced among individuals with higher BMIs.

### 2.7. Long-Chain Fatty Acids (FAs) Lead to a Significant Decline in Primary PT Variables

**Figure 7:**
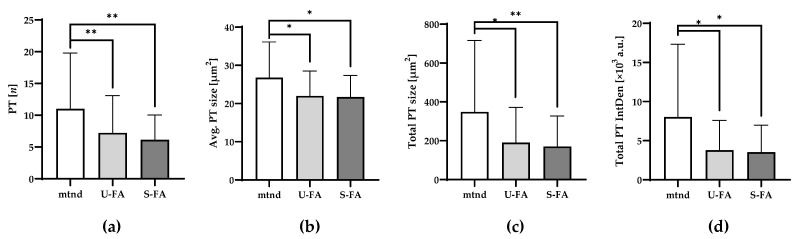
Fatty acids (FAs) decrease PT expansion in human skeletal muscle tissue in vitro. (**a**) Skeletal muscle tissue sections from all participants (*n* = 12) were embedded following in vitro maintenance without (mtnd) or supplemented with unsaturated FA (U-FA) or saturated FA (S-FA). After IF staining and imaging, the PT variables (*y*-axes) of the mtnd group were compared with those of the respective U-FA- or S-FA-treated samples. PT variables, number (**a**), average size (**b**), total size (**c**), and total IntDen (**d**) are presented. A paired *t* test or Wilcoxon signed-rank test was used to assess the significance of the detected differences. *p* ≤ 0.05 (*), *p* ≤ 0.01 (**).

Previously, we examined the effects of long-chain FA–induced metabolic signaling and reported a significant increase in metabolism and TRM polarization in human skeletal muscle tissue [33]. To further assess the potential link between metabolic conditions and PT characteristics, we incubated additional sections from all the samples with either U-FAs or S-FAs and compared them with samples maintained without FAs (mtnd), as described in Section 2.1 and Section 2.5. As shown in Figure 7, both U-FAs and S-FAs accelerated the decrease in primary PT imaging variables, including total number (Figure 7a), average size (Figure 7b), total size (Figure 7c), and IF signal density (Figure 7d). These reductions ranged from approximately 20% to 50%, with particularly strong statistical significance despite the modest sample area. Notably, we did not detect a difference between U-FA- and S-FA-mediated effects. Moreover, other spatial PT variables, such as FI and CI, were marginally affected by U-FA or S-FA (Supplementary Materials Figure S4). These results suggested that changing metabolic conditions may have a stronger effect on SV2 and 2H3 expression than on spatial PT fragmentation. Moreover, FA-mediated metabolic stimulation improves PT integrity and reverts PT expansion in vitro.

## 3. Discussion

Investigating the structural and functional plasticity of human neuromuscular junctions (NMJs) is essential for elucidating the pathogenic processes underlying neurodegenerative disorders and for differentiating these mechanisms from those associated with phenotypically similar conditions such as sarcopenia and age-related decline. Presynaptic modulation of NMJs is a fundamental mechanism for regulating synaptic transmission and shaping overall synaptic function. The present study focused on presynaptic terminals (PTs) in a human skeletal muscle model and defined a set of key variables for evaluating PT IF images across study participants and experimental conditions. The data depict PT characteristics and their alterations in response to age, metabolic signals, and extended muscle inactivity. Thus, the applied human skeletal muscle model enables a more detailed investigation of NMJ function and cross-talk with regulatory cell populations within the native three-dimensional microenvironment of skeletal muscle tissue. These features offer new opportunities to examine NMJ dynamics in pathological contexts, identify potential therapeutic targets, and assess candidate compounds aimed at delaying or preventing the onset of neuromuscular impairment.

The present study revealed significant associations between human PT remodeling, aging, and inflammatory factors. These findings were supported by a comprehensive set of quantitative variables that enabled detailed comparisons across large numbers of PT images. PT number, average size, total size, and IF intensity—reflecting increased expression of SV2 and 2H3—were positively correlated with age (Figure 3). Notably, aging was not associated with spatial PT formation, such as the fragmentation index (FI), circularity index (CI), or solidity index (SI). This finding is consistent with early histological studies in which silver and cholinesterase staining in longitudinal muscle sections from human cadavers; these studies revealed age-dependent changes in NMJ morphology and AChR distribution [39] and substantial reinnervation failure in aged human muscle [40]. Studies in which simultaneous staining of pre- and postsynaptic components in frozen sections was employed have revealed age-related increases in nerve terminal and endplate area; however, concluded that the overall architecture of the human NMJ remains largely preserved across the lifespan [11]. These discrepancies may stem from differences in sample preservation and staining techniques, signal overlap between pre- and postsynaptic markers, or variations in evaluation criteria.

Collectively, our results support the hypothesis that impaired muscle function—whether due to disease, aging, or injury—is accompanied by substantial PT expansion and fragmentation. In the present study, immediate postsurgical samples were considered controls because they were preserved within 90 min of dissection from the donor’s muscle. However, potential transient effects of surgical trauma or local inflammation cannot be determined or excluded. By contrast, the differences observed between immediate postsurgical controls and mtnd samples reflect long-term effects on muscle tissue after 11 days. The prolonged in vitro maintenance of muscle tissue and aging had similar effects on increasing PT number, size, and integrated density (Figure 5). Unlike age, extended maintenance reduced PT circularity but increased PT fragmentation and expansion, indicating advanced spatial remodeling. Furthermore, elevated BMI, as a metabolic precondition, is correlated with increased PT fragmentation and decreased PT circularity, indicating that it promoted PT remodeling in vitro (Figure 6). Importantly, exposure of muscle tissue to long-chain FAs—which promote metabolic activity in skeletal muscle [32,34,41]—partially reverses PT remodeling in vitro, restoring PT parameters to the levels observed immediately after surgery. Moreover, our data revealed no significant differences between the effects of U-FA and S-FA on PT remodeling (Figure 7). Previously, we reported that U-FA and S-FA equally activate anti-inflammatory CD206+ TRMs in human skeletal muscle [33]. In fact, numerous human studies acknowledged the link between anti-inflammatory and pro-metabolic mediators in skeletal muscle tissue [42]. These observations align with recent human biopsy data demonstrating that metabolic myopathies and impaired mitochondrial function are associated with primary NMJ remodeling independent of overt muscle damage [43]. Thus, it is conceivable that long-chain FAs promote metabolic activity and establish an anti-inflammatory milieu close to NMJs. However, the potential mechanistic link between these FA-mediated effects remains to be determined. Likewise, it is still unclear whether PT remodeling precedes or follows skeletal muscle dysfunction; further refinement of our experimental model may help clarify the underlying molecular events and causal relationships involved. Importantly, this model overcomes a major limitation of retrospective human biopsy studies by enabling temporal analyses of cells and molecular processes within the native microenvironment of human NMJs.

Studies in transgenic mouse models have implicated tissue-resident macrophages (TRMs) in peripheral nerve injury and repair processes [44,45,46]. In a rat model of familial amyotrophic lateral sclerosis (ALS), CD11b-positive macrophages were observed close to postsynaptic AChRs; however, the high variability among samples prevented definitive conclusions regarding their significance [44]. In our study, we analyzed macrophages as integral components of the microenvironment surrounding PTs. Although our analysis was restricted to CD206 and CD80 markers, the presence of other macrophage subtypes is highly plausible. Previously, we identified 6 different subtypes of macrophages in human skeletal muscle tissue samples [33,34], reinforcing the concept that different subtypes of macrophages constitute stable elements of the human NMJ niche. Notably, we observed no significant correlations between the abundance of NMJ-associated TRMs abundance and donor age or BMI (*p *≥ 0.1091 and *p *≥ 0.3228, respectively). Expanded molecular and phenotypic profiling of macrophages residing near NMJs will be essential for elucidating the mechanistic links between local inflammation and neuromuscular function, ultimately clarifying their roles in human health and disease.

By focusing exclusively on PT markers and applying an expanded set of quantitative variables, our analysis revealed age-related PT plasticity that may have been overlooked in previous work. Moreover, our findings suggest that inflammatory (RANTES) and neurotrophic (NGF) beta factors may augment PT expansion. Our data indicate, that RANTES expression significantly increased with PT number, size, and expression in human skeletal muscle tissues. Initially, RANTES was shown to direct the movement of immune cells in the peripheral immune system [47]. In the central nervous system; however, elevated RANTES expression—by microglia and astrocytes in response to inflammation—was linked to accelerated cognitive decline [47]. NGF beta is involved in the regulation of neuronal growth and repair [48]. Our data indicate for the first time that NGF beta expression in human skeletal muscle tissue increases with PT average and total size as well as with age and BMI, suggesting that NGF beta is involved in PT remodeling.

Several limitations should be considered when interpreting this study model. The first limitation is the small number of participants. Thus, the identified correlations need to be further verified in future investigations, including larger and more diverse cohort of participants. Second, our study monitored the dynamic changes into PT structure rather than the functional assessment of NMJs over time. Although, this limitation generally applies to all histological studies, future research may develop morphological or functional assessment tools which reveal PT function, such as noninvasive measurements of action potentials using electrodes embedded in culture plates. Previous studies suggested robust relationships between synaptic morphology and function in mice [11,49]. A recent study of 130 human cases reported that NMJ transmission measured by intramuscular electromyography corresponds to NMJ stability assessed by the overall tissue level of agrin pathway downstream components [50]. Similarly, the measured protein levels in our study reflect overall tissue expression rather than NMJ-specific protein expression. The identification of locally expressed proteins that directly contribute to PT remodeling requires advanced spatial expression analyses. Moreover, conventional protein expression analyses of maintained samples in our study were challenging to conduct because of the limited amount of tissue. Third, assessing the three-dimensional architecture of PTs from two-dimensional images represents an inherent limitation—common to all human and animal NMJ study models—although this constraint was partially mitigated by the extended analysis of more than 3530 PTs in our study. Fourth, the histological PT assessments relied on two previously established 2H3 and SV2 markers and the best quality secondary antibody in preliminary experiments. By primary comparison of different antibodies, however, we noticed significant differences in signal interference and reproducibility, highlighting the limitations of immunofluorescence staining. Thus, future human longitudinal investigations and development of efficient NMJ 3D-image reconstruction tools will be needed to address the potential link between the morphological findings and NMJ function.

In conclusion, the native skeletal tissue model presented here represents the first attempt to validate and characterize the temporal plasticity of human PTs under controlled experimental conditions. Although age and BMI largely determine the baseline properties of PTs, latent skeletal muscle tissue in vitro appears to facilitate their spatial fragmentation and expansion. Thus, our current findings may have broad and direct implications for early differential diagnosis of muscle atrophy and neuromuscular disorders. Future studies should integrate additional functional components of human NMJs, including the synaptic extracellular matrix, the postsynaptic apparatus, and relevant supporting cell types. Continued investigations of these molecular and structural adaptations will be essential for clarifying the mechanisms underlying age-associated neuromuscular decline and may ultimately inform the development of therapeutic strategies to prevent sarcopenia, frailty, and related neuromuscular disorders. Moreover, our model can serve as a native human functional platform for the discovery of novel therapeutics aimed at repairing, protecting, or enhancing neuromuscular junction (NMJ) function, particularly in myasthenia gravis (MG), Lambert-Eaton myasthenic syndrome (LEMS), congenital myasthenic syndromes (CMS), or amyotrophic lateral sclerosis (ALS).

## 4. Materials and Methods

### 4.1. Tissue Specimens

Skeletal muscle tissue specimens were obtained from patients (*n* = 12) undergoing surgery at the Clinic for Orthopedics, Trauma, and Reconstructive Surgery at RWTH Aachen University Hospital, Germany. Immediately after excision, the surgeon placed each sample into a sterile container and transferred it directly to the laboratory. The study was approved by the Medical Ethics Committee of RWTH Aachen University prior to sample collection (No. EK206/09). All participants and surgeons provided written informed consent, with the documents archived in the Centralized Biomaterial Bank (cBMB) at RWTH Aachen University Hospital.

### 4.2. Quantification of Neurotrophic Factors in Tissue Extracts

Tissue extracts were prepared from 2.5 mg of tissue using two preconfigured ProcartaPlex assay (EPX040-15828-901 and EPX160-12176-901; Thermo Fisher Scientific, Waltham, MA, USA) according to the manufacturer’s instructions. The assay panel quantified four human neurotrophic factors: glial cell line-derived neurotrophic factor (GDNF), ciliary neurotrophic factor (CNTF), brain-derived neurotrophic factor (BDNF), and nerve growth factor-beta (NGF beta) as well as 16 inflammatory factors: IFN alpha, IL-1 alpha, IL-1RA, IL-7, IL-8 (CXCL8), IL-15, IL-31, TNF beta, Eotaxin (CCL11), GRO alpha (CXCL1), IP-10 (CXCL10), MCP-1 (CCL2), MIP-1 alpha (CCL3), MIP-1 beta (CCL4), RANTES (CCL5), SDF-1 alpha. For each analyte, the mean of four independent measurements was used for final concentration values and statistical analyses.

### 4.3. Maintaining and Treatment of Human Tissue Specimens

All Tissue samples were cut into uniform 18-mm^3^ sections. As an initial reference, one section was fixed immediately after dissection in 4% formaldehyde for 24 h (Otto Fischar GmbH, Saarbrücken, Germany). The remaining sections were separately embedded in a gel medium containing 1% low-melt agarose (Carl Roth GmbH, Karlsruhe, Germany), 10% fetal bovine serum (PAN-Biotech GmbH, Aidenbach, Germany), and 100 U/mL penicillin–100 U/mL streptomycin (PAN-Biotech GmbH) in DMEM (Biological Industries, Kibbutz Beit-Haemek, Israel) at 40 °C.

For FA-treatments, unsaturated FAs (FA 1208 and 1024; Biotrend Chemikalien GmbH, Cologne, Germany) or saturated FAs (1014 and 1020; Biotrend Chemikalien GmbH) were dissolved at a 1:2.5 ratio in 10% bovine serum albumin (BSA) in PBS and added to the gel medium to a final concentration of 50 µM. After solidification at room temperature, DMEM/F-12 medium (Gibco, Thermo Fisher Scientific, Waltham, MA, USA) containing 10% fetal bovine serum and 100 U/mL penicillin–100 U/mL streptomycin was layered on top of the gel. Embedded tissues were maintained at 37 °C in 5% CO_2_. After 9 or 11 days, the medium was removed, and tissue samples were carefully extracted from the gel and fixed in 4% formaldehyde (Otto Fischar GmbH) for 24 h.

### 4.4. Staining with Hematoxylin and Eosin (HE Staining)

Tissue samples were dehydrated through ascending ethanol concentrations (70%, 96%, and 100%) and embedded in paraffin. Paraffin blocks were sectioned at 5 µm using a SLIDE4003E microtome (pfm Medical, Cologne, Germany), and sections were mounted onto glass slides. Deparaffinization and hematoxylin–eosin (H&E) staining were performed using an automated slide stainer (Gemini, Thermo Fisher Scientific, Waltham, MA, USA). Slides were deparaffinized, stained with hematoxylin for 5–10 min, rinsed in warm water for 10 min, stained with 0.3% eosin for 5 min, and washed with distilled water. Finally, slides were dehydrated through ascending ethanol concentrations (70%, 96%, and 100%), cleared in xylene, and covered with glass cover slips.

### 4.5. Immunofluorescence (IF) Staining of Human Macrophage Markers

Paraffin-embedded tissue sections were deparaffinized and subjected to heat-induced antigen retrieval in citrate buffer (pH 6.0) for 30 min. After cooling in water, slides were rinsed twice in PBS containing 0.1% Tween-20 (9127.1, Carl Roth) and subsequently blocked in UltraCruz Blocking Reagent (Santa Cruz Biotechnology, Dallas, TX, USA) for 60 min. Primary antibodies against presynaptic terminal markers SV2 (1:100; AB2315387; DSHB) and 2H3 (1:100; AB531793; DSHB) were diluted in UltraCruz Blocking Reagent (Santa Cruz Biotechnology) and applied overnight at 4 °C. Next day, primary antibodies against macrophage markers CD80 (1:1000; ab134120, Abcam, Cambridge, UK) and CD206 (1:100; PA5-101657, Thermo Fisher Scientific) were diluted in UltraCruz Blocking Reagent (Santa Cruz Biotechnology), added to the reaction, and incubated at 4 °C for another 24 hrs. Following washes, slides were incubated for 60 min with secondary antibodies—anti-rabbit IgG488 (1:100; ab150081, Abcam), anti-mouse IgG594 (1:200; ab150120, Abcam) diluted in UltraCruz Blocking Reagent (Santa Cruz Biotechnology). After an additional wash step, nuclei were counterstained with 0.1% DAPI (D9542, Sigma-Aldrich, St. Louis, MO, USA) in PBS for 5 min. Slides were mounted with Immu-Mount (9990402, Thermo Fisher Scientific) and sealed with glass coverslips.

### 4.6. Imaging and Image Analysis

Microscopy and imaging of HE and IF images were performed using an automated microscope (DM6000B; Leica Microsystems, Wetzlar, Germany) equipped with an integrated digital camera. Fluorescent images were captured using the following filter sets: 340–380 nm for DAPI, 450–490 nm for anti-rabbit IgG488, and 590 nm for anti-mouse IgG594. Image processing and merging were conducted using Diskus software (version 10; Leica). For each individually stained section, three to seven non-overlapping cross-sectional muscle fields (each covering 0.252 mm^2^) containing neuromuscular junctions were acquired at 20× magnification using identical exposure settings across all samples, strictly excluding areas other than skeletal muscle fibers. Quantitative analysis of presynaptic terminals (PTs) was performed independently by two blinded investigators. Both investigators verified isolated single PTs using transmitted light microscopy, defined and marked regions of interest (ROIs), and subsequently merged these ROIs for detailed IF signal analysis. ROIs were then analyzed in Fiji/ImageJ v1.54p, accessed on November 20, 2024 (National Institutes of Health, Bethesda, MD, USA) using the software’s built-in tools to obtain morphometric and intensity variables. Raw data were exported, calculated, and summarized in Microsoft Excel 365 (version MSO; Redmond, WA, USA).

### 4.7. Statistical Data Analysis

The distribution of continuous variables was assessed using the Shapiro–Wilk test. Protein concentrations were transformed using log1p (i.e., ln (1 + x)) and corrected for inter-batch differences by linear regression (log-transformed concentration~batch); the resulting batch-corrected log values were used for subsequent analyses. Correlations between batch-corrected log protein expression levels and clinical or morphometric characteristics were analyzed using Pearson’s correlation or Spearman’s rank correlation, with Benjamini–Hochberg false discovery rate (FDR) correction. Changes in normalized values were evaluated using one-sample *t*-tests or Wilcoxon signed-rank tests, and group differences were assessed using paired *t*-tests or Wilcoxon signed-rank tests, as appropriate. All statistical analyses were performed using R (version 4.4.2; R Foundation for Statistical Computing, Vienna, Austria) with the tidyverse package (version 2.0.0) and GraphPad Prism (version 10.2.3; GraphPad Software, San Diego, CA, USA).

## Figures and Tables

**Table 1:** Donor characteristics.

Participants	Sex	Age (Years)	BMI (kg/m^2^)	Type 2 Diabetes (T2D)	Locations
P1	F	67	46.90	Yes	1
P2	F	69	24.8	No	2
P3	F	57	22.3	No	4
P4	M	67	28.6	No	3
P5	F	22	20.3	No	3
P6	M	22	20.20	No	3
P7	M	69	39.5	No	3
P8	F	56	19.4	No	4
P9	M	39	24.4	No	2
P10	M	37	30.4	No	4
P11	F	84	27.5	No	2
P12	M	36	29.1	No	2

Participants (P); female (F); male (M); pronator quadratus muscle (1); vastus lateralis muscle (2); multifidus muscle (3); obliquus externus abdominis muscle (4).

**Table 2:** PT variables.

Index	Formula	Definition
PT number		PT number/0.252 mm^2^
Avg. PT size		Average PT area size
Total PT size		Total PT area size/0.252 mm^2^
IntDen/PT		Integrated Density/PT
IntDen/PT size		Integrated Density normalized to PT size
Total IntDen		Total Integrated Density/0.252 mm^2^
Fragmentation index	$FI=1-\frac{1}{N}$,	N: Number of clusters; FI range: 0.0–1.0
Circularity index	$CI=4\pi \times \frac{Area}{{Perimeter}^{2}}$	CI range: 0.0–1.0
Roundness index	$RI=\frac{4\times Area}{\pi \times {Axis}_{Major}^{2}}$	RI range: 0.0–1.0
Aspect ratio	$AR=\frac{{Axis}_{Major}}{{Axis}_{Minor}}$	AR range: 0.0–1.0
Solidity index	$SI=\frac{{Area}_{actual}}{{Area}_{Convex}}$	SI range: 0.0–1.0

Presynaptic terminal (PT).

**Table 3:** TRMs located close to PTs in human skeletal muscle tissue.

TRM	Examined PTs	Proximate to TRM *	
	[n]	[n]	[%]
CD80	320.7	16.76	5.23
CD206	185.65	16.45	8.86
CD80 or CD206	506.36	33.21	6.56

* within a radius of ≤18 µm.

## Data Availability

The original contributions presented in this study are included in the article/Supplementary Materials. Further inquiries can be directed to the corresponding author.

## Supplementary Materials

The following supporting information can be downloaded at: https://www.mdpi.com/article/10.3390/ijms27041928/s1.

## Abbreviations

The following abbreviations are used in this manuscript:

NMJ	Neuromuscular junction
PT	Presynaptic terminal
SV2	Synaptic vesicle protein
2H3	Neurofilament
AChR	Acetylcholine receptor
ECM	Extracellular matrix
ALS	Amyotrophic lateral sclerosis
TRM	Tissue-resident macrophage
T2D	Type 2 diabetes
BMI	Body mass index
ctrl	Control
mtnd	Maintained
Avg.	Average
FI	Fragmentation index
CI	Circularity index
SI	Solidity index
