# Supplementary material for: Spatiotemporal Remodeling of Presynaptic Terminals in Human Neuromuscular Junctions
Source: Int J Mol Sci. 2026 Feb 17;27(4):1928. doi: 10.3390/ijms27041928 (PMC12940820; doi:10.3390/ijms27041928)
Supplement: Supplementary file 1 [file ijms-27-01928-s001.zip › ijms-4065597-supplementary.pdf]

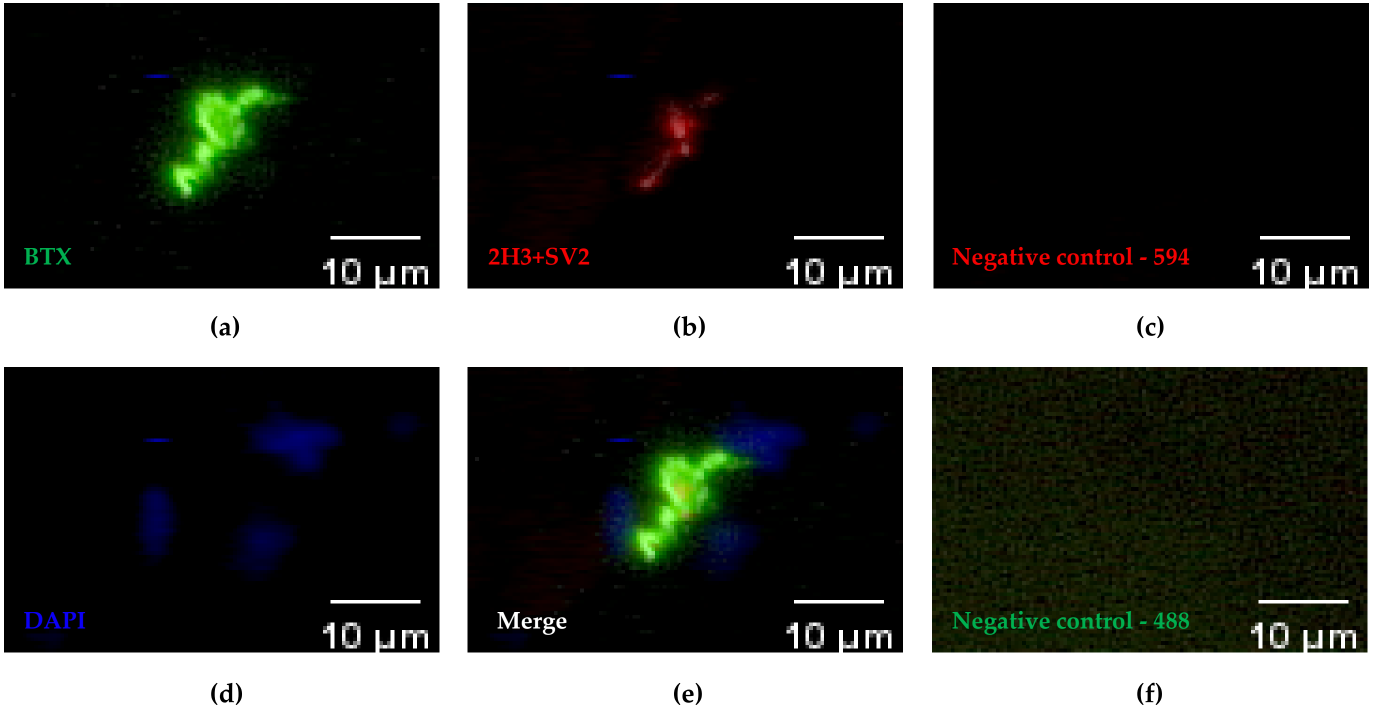

Figure S1: Representative IF images of immediate postsurgical skeletal muscle tissue from a male participant (34 years). The frozen tissue sections were subjected to immunofluorescence staining for 2H3 and SV2 and Fluor 594-conjugated secondary antibody (red) together with DAPI (blue) and Fluor 488-conjugated Bungarotoxin (green). Images of the same field were obtained using DyLight 488 (a and f) or 594 (b and c) filters and then merged to facilitate clearer recognition of labeled structures (e). Staining without antibodies against 2H3, SV2, and bungarotoxin (c and f) served as negative controls. The white scale bars (lower right) indicate 10 µm, and IF images correspond to tissue fields of 50 µm × 30 µm.

Table S1: The relative fold-change of PT image variables of all the participants

Patients	PT number	Ave. PT size	PT size sum	IntDen per PT	IntDen/PT size	IntDen sum	FI	CI	RI	AR	SI
P1	2.37	1.80	4.39	2.12	1.26	4.95	1.60	0.64	0.99	1.06	0.84
P2	0.92	1.33	1.30	1.50	1.15	1.48	1.40	0.74	0.99	0.96	0.82
P3	1.48	0.84	1.44	1.05	1.10	1.72	1.12	0.95	0.84	1.05	0.98
P4	1.64	1.16	1.82	1.39	1.05	2.23	1.28	0.84	1.09	0.86	0.96
P5	1.79	1.65	3.30	1.64	1.03	3.05	1.19	0.78	0.94	1.04	0.84
P6	2.51	1.01	1.93	0.53	0.63	1.09	1.04	1.03	1.64	0.64	1.19
P7	0.97	1.27	1.33	1.44	1.10	1.60	1.46	0.83	0.93	1.03	0.99
P8	1.13	1.22	1.33	1.27	1.14	1.3	0.73	1.10	0.92	1.15	1.13
P9	0.69	0.93	0.59	0.97	1.07	0.71	0.82	0.86	1.00	1.07	0.90
P10	1.73	1.56	3.05	2.19	1.33	4.13	1.15	0.78	1.02	1.01	1.10
P11	1.75	1.11	1.91	0.97	0.83	1.79	1.04	0.90	1.00	0.98	1.00
P12	0.31	0.73	0.24	0.67	0.73	0.22	1.84	0.67	0.76	1.43	0.73

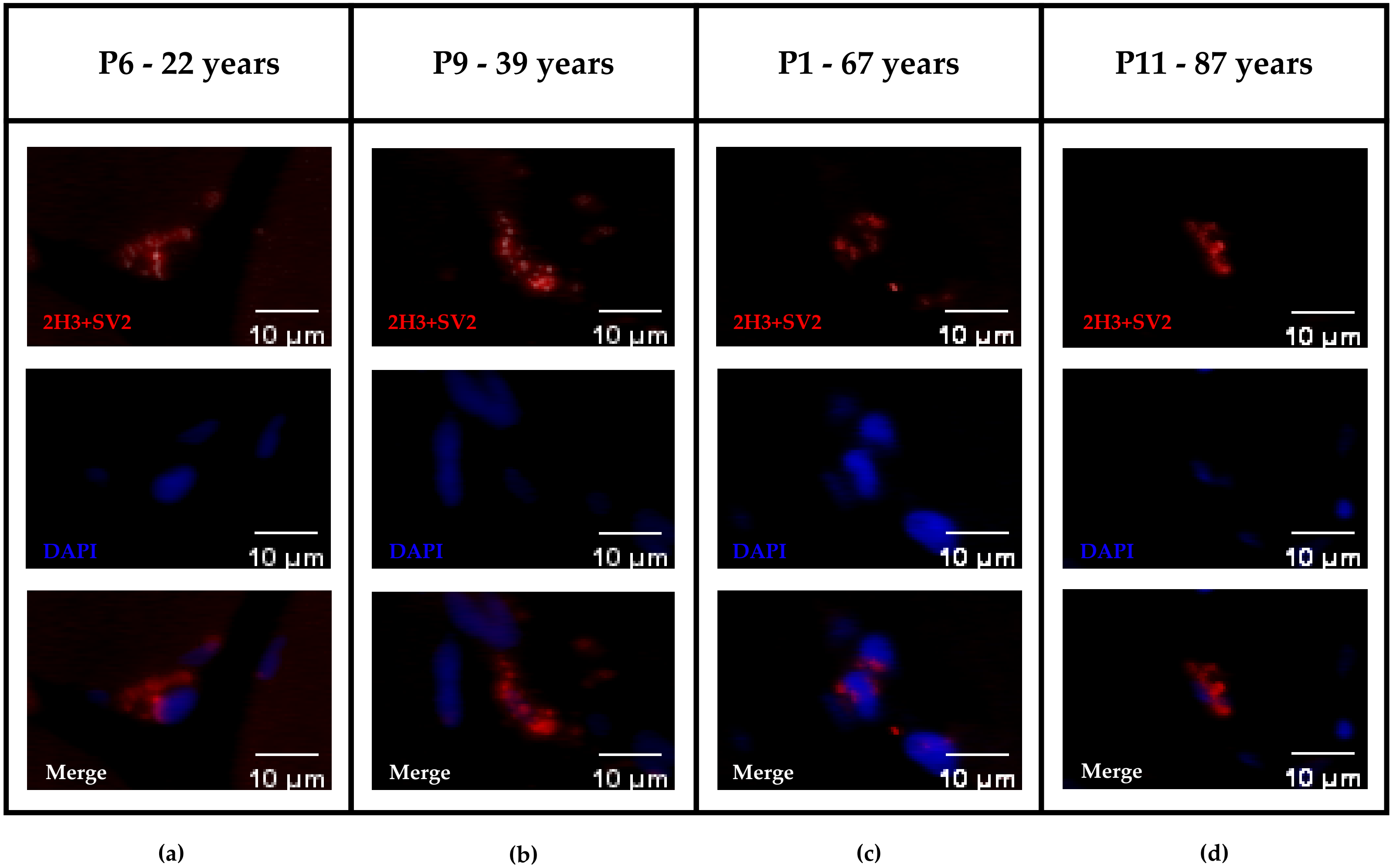

Figure S2: Representative IF images of neuromuscular junctions from human skeletal muscle samples across different age groups (22, 39, 67, and 87 years). PTs were labeled with antibodies against 2H3 and SV2 (red), and nuclei were counterstained with DAPI (blue). Merged images from the same field of view are shown. Scale bars indicate 10 µm.

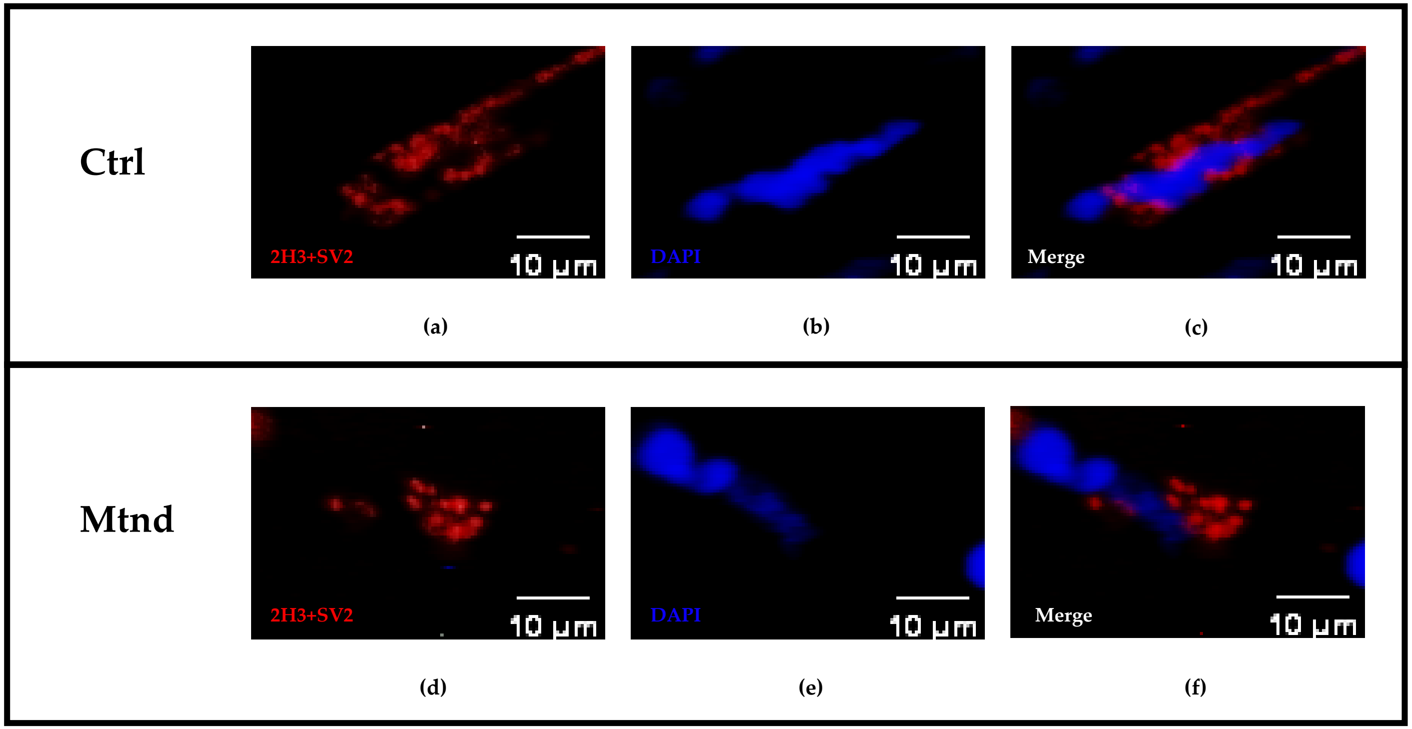

Figure S3: Representative IF images of NMJs under control and mtnd experimental conditions. Images of an 84 years old participant (P11) are presented. IF images correspond to tissue fields of 50 µm × 30 µm.

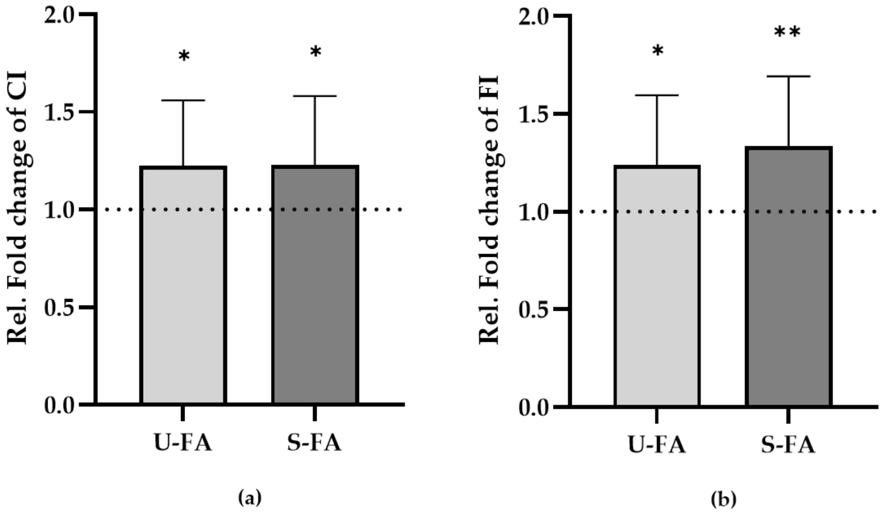

Figure S4. FAs do not affect PT image variables, CI and FI. **(a)** Skeletal muscle tissue sections from all participants (n = 12) were embedded following in vitro maintenance without (mtnd) or supplemented with unsaturated FA (U-FA) or saturated FA (S-FA). After IF staining and imaging, the PT variables (y-axes) of the mtnd group were compared with those of the respective U-FA- or S-FA-treated samples. Relative fold change of the PT variables, CI (**a**) and FI (**b**) are presented. A paired *t* test or Wilcoxon signed-rank test was used to assess the significance of the detected differences. $p \leq 0.05$ (*), $p \leq 0.01$ (**).
